# How Do Courts Set Health Policy? The Case of the Colombian Constitutional Court

**DOI:** 10.1371/journal.pmed.1000032

**Published:** 2009-02-17

**Authors:** Alicia Ely Yamin, Oscar Parra-Vera

## Abstract

Alicia Ely Yamin and Oscar Parra-Vera discuss the case of Colombia, where a recent constitutional court decision demonstrates the involvement of the court in protecting fundamental rights to health.

On July 31, 2008, the Constitutional Court of Colombia (the Court) handed down a decision (T-760/2008) that ordered a dramatic restructuring of the country's health system [1]. The judgment came as the culmination of a wave of litigation to enforce the right to health, with tens of thousands of health rights cases before the Colombian courts each year [2]. Since 1992, the Court has staunchly upheld rights to access and treatment in the context of a highly neoliberal state, and has not shied away from decisions with considerable resource implications.

Colombia is a striking example of how broader regional and global trends can have an impact on judicial enforcement of claims for health goods and services [3–6]. However, there is a wide-ranging debate in public health circles about the appropriateness and impact of such judicial interventions on health policy and health equity [5]. Critics question, for example, whether judicial activism distorts priority-setting and undermines the role of administrative and legislative bodies [3–5,7].

Although it is too early to judge the implementation of the July 2008 decision, the sweeping 411-page judgment reaffirms that courts can enforce access to health goods and services as a matter of fundamental rights, even when there are substantial resource implications. It further indicates that courts can creatively define their role in health priority-setting.

## Background

Colombia is a middle-income country with a per capita gross domestic product of US$7,304 (purchasing power parity 2005), and a Gini coefficient of 58.6, reflecting a level of economic inequality that is among the highest in the world [8]. Almost two-thirds of the 46 million population live below the poverty line [8]. In Colombia, a tradition of creating democratic institutions has coexisted with a reality of authoritarianism and enormous political violence, notably the 50-year-old armed conflict [9].

The 1990s brought dramatic, albeit contradictory, changes to both the judicial and health systems. The 1991 Constitution created a Constitutional Court, together with mechanisms such as the *tutela* (protection writ) to protect individual rights, and greatly enhanced the public's access to the courts through unfettered standing and lack of procedural requirements [10].

In 1993, the Colombian health care system underwent a major reform, with the passage of Law 100. In keeping with neoliberal ideas for sectoral adjustment in the early 1990s, Law 100 shifted government subsidies from supply to demand and created a “competitive surrogate model” that used public and private insurers as surrogates to purchase health care for insured patients, with the goal of improving efficiency [11]. It also established a two-tier system of benefits: (i) the contributory regime (*Plan Obligatorio de Salud*, or POS) for those formally employed or earning more than twice the minimum wage, and (ii) the subsidized regime (*Plan Obligatorio de Salud Subsidiado*, POSS), which includes approximately one-half of the benefits in the contributory regime [11–13].

## History of Judicial Enforcement of the Right to Health

Although coverage has increased since 1993, the Colombian health system has been widely criticized—efficiency and quality gains have generally not materialized, and patients have increasingly turned to the courts to secure treatments and services [11–18]. Between 1999 and 2005, the Human Rights Ombuds Office calculates that 328,191 *tutelas* were presented relating to the right to health; in approximately 80% of those cases the *tutela* was granted [14]. Unlike the common law system, the vast majority of these cases resolved only the dispute in the individual case before the court. Nevertheless, the sharply increasing numbers of *tutelas*—approximately 90,000 per year by 2008—are alarming [2,14,19].

According to the jurisprudence of the Constitutional Court, which reviews *tutela* judgments from courts throughout the country, the right to health is enforceable for plaintiffs unable to afford care: (i) when there is an inextricable relationship with “fundamental rights,” including the right to life, such that if the right to health were not protected immediately it would result in the violation of these latter rights; (ii) when the case involves a person or group of people in especially vulnerable circumstances, such as children, pregnant women, or the elderly; and (iii) when the health good or service at issue is included in the POS/POSS, which the Court has taken to define a minimum core content of the right to health.

Based on these criteria, the Court has ordered the provision of a wide range of goods and services, including antiretrovirals, costly cancer medications, and even the financing of treatment of patients abroad when appropriate treatment was unavailable in Colombia. Likewise, the Court has exercised judicial control over the procedures used to determine benefits in the POS, as well as those used to determine beneficiaries of subsidized care [2,14,19].

In cases where the Court orders provision of care not included in the POS/POSS, a government Solidarity and Guarantee Fund (*Fondo de Solidaridad y Garantía*, FOSYGA) is required to reimburse the provider for expenses incurred, which has resulted in the majority of FOSYGA's budget being dedicated to the implementation of *tutelas* [20]. Compliance with individual judgments, as well as broader orders, has often been slow and irregular, largely due to the inordinately complex procedures through which the state reimburses the entities providing the care ordered by the courts [1,2,14,19].

However, there is no question that use of *tutelas*, and the judgments of the Court in particular, have had a dramatic impact on prospective health policy-making and budgeting and the enforcement of existing laws [20]. For example, judgments from the Court have led to the modification of the POS to include viral load tests for HIV (T-654/2004); similarly, when the Court found proposed budget cuts to the POSS to constitute impermissible retrogression (backsliding), the budget was revised (C-1165/2000).

On the other hand, the overwhelming preponderance of cases in which courts have enforced the right to health in the country as a whole relate to health goods and services that the state had already agreed to provide. A recent study by the National Human Rights Ombuds Office found that between 1999 and 2005 approximately 89% of the surgeries, 93% of the treatments, and 84% of the procedures that were petitioned for using *tutelas* were already included in the POS or POSS. These findings suggest a health system with little capacity for internal regulation, where judicial recourse has become, according to a 2008 report from the Attorney General's Office, an “escape valve” [14,19].

## A Landmark Decision

T-760/2008 collects 22 *tutelas* (20 brought by individuals and two brought by insurance companies), which were selected in order to illustrate systemic problems in the health system that have led to the overuse of the *tutela*. The judgment resolves not only the 22 cases before the court, but also calls for the transformation of the entire system. The Court asserted this structural approach was necessary because “the organs of government responsible for…the regulation of the health system have not adopted decisions that guarantee the right to health without having to seek recourse through the *tutela*” [1].

Indeed, 20 of the 22 cases relate to principles that the Court has repeatedly established, but that health care providers and insurance companies continue to fail to assimilate in their policies due to a failure of oversight and regulation [1]. Among those cases were restrictions on access to care stemming from inappropriate transfers of administrative costs to patients and failures to make access effective (e.g., by ignoring transportation needs), as well as restrictions on access to care “necessary for the adequate development of a child” (cochlear implant) and for catastrophic conditions. The Court reiterates and clarifies its ample jurisprudence regarding the enforceability of the right to health in such cases and also calls for measures to reduce recourse to *tutelas*, including increased authority for the scientific-technical committees of insurance companies [1].

Some cases also addressed the freedom to choose among providers and the process for determining whether a given service was included in the POS (e.g., intraocular lens). Further, two cases were taken from insurance companies regarding reimbursement for services not included in the POS, and adjustments in the regulations regarding reimbursements. The Court analyzes these cases in terms of state failures to protect the right to health. It calls for: (i) transparency in determinations of POS benefits as well as institutional performance audits to inform users about the performance of different providers and insurance entities, in particular in relation to the numbers of *tutelas* their affiliated patients must bring to secure care; and (ii) measures to facilitate execution of *tutelas*, as well as the adoption of a contingency plan to ensure appropriate and timely reimbursements in the event of costs associated with care not covered under the POS/POSS [1].

The Court goes even further, however, calling for restructuring of the benefit plans themselves. In keeping with what the legislature itself ordered in 2007 (Law 1122), the judgment directs the National Commission for Health Regulation to immediately and on an annual basis comprehensively update the benefits included in the POS/POSS through a process that includes “direct and effective participation of the medical community and the users of the health system” [1]. Citing the government's failure to take any steps toward a unification of plans since the adoption of Law 100, the Court further orders the appropriate executive agencies to unify the benefit plans (POS and POSS), initially for children and later for adults, in the latter case progressively and taking into account financial sustainability, as well as the epidemiological profile of the population. The process of devising a unification plan is to be participatory, transparent, and evidence-based, and must include relevant indicators and benchmarks [1].

The judgment calls upon the government to adopt deliberate measures to progressively realize universal coverage by 2010, and sets various compliance deadlines in 2008 and 2009 [1]. Although it is too early to judge implementation of the July decision, in August, the Minister of Social Protection stated publicly that a timetable for the unification of the POS and POSS was being drawn up, but estimated that it would cost as much as 6.5 trillion pesos (approximately US$3.25 billion), which he asserted “the State does not have” [21]. At the same time, the Minister reported that immediate orders, such as provisions for the expanded authority of scientific-technical committees, were being implemented [22]. In September, a group of senators formally sent the Ministry of Social Protection a comprehensive list of questions regarding Law 100, Law 1122 of 2007, and the plans for follow-up on T-760 [23].

## Discussion

The Court's decision is notable in many respects, not least of which is its explicit adoption of the right to health framework set out by the United Nations Committee on Economic, Social and Cultural Rights (UN ESC Rights Committee) and clarified through the work of the first Special Rapporteur on the Right to Health [24]. In keeping with the UN ESC Rights Committee's interpretation of the right to health, the Court: (i) elaborates on the multiple dimensions of state obligations that flow from the right to health, and how oversight is essential to *protecting* the right to health as well as to accountability; (ii) reiterates that the state is responsible for adopting deliberate measures to achieve progressive realization of the right to health and that retrogression (backsliding) is generally impermissible; and (iii) asserts that the right to health calls for transparency and access to information, as well as for evidence-based planning and coverage decisions based on participatory processes [1,24].

Further, the Court reaffirms its jurisprudence distinguishing an essential minimum core of the right to health based on the POS/POSS, which is immediately enforceable, and other elements that are subject to progressive realization, taking into account resource constraints [1,24]. This approach contrasts with, e.g., the South African Constitutional Court, which has rejected the notion of a minimum core that can be enforced without regard to resources, and has instead focused on “reasonableness” [25].

However, the Colombian Court is not alone in enforcing programmatic claims for health services that have significant economic impacts. Across Latin America—a region characterized by rights-rich constitutions, high social exclusion, and systemic failures of representation by the political branches of government—and especially in Argentina, Colombia, Brazil, and Costa Rica, courts have enforced access to HIV/AIDS medications as well as to a wide range of other treatments and services. In Costa Rica, the first ten years of operation of the Sala IV, as the Constitutional chamber of the Supreme Court is known, witnessed an increase in the *amparo* (similar to the Colombian *tutela*) caseload from less than 1,000 cases to over 11,000 in 2001, a substantial and growing fraction of which relate to health claims [6]. In Brazil, thousands of court cases have been brought since 1992 relating to access to medications—many of which are highly costly and not included in Brazil's national health plan—resulting in distortions of the health budget [3,5]. In Argentina, as in Colombia, the Supreme Court has gone beyond issuing relief in individual cases to examining policies and regulations in the health sector, for example, ordering medical coverage for persons with HIV/AIDS and other vulnerable groups, as well as enforcing the extension of medical coverage under prepaid health plans, and granting interim relief to assure access to care in emergency situations [26].

Although it is too soon to say, the structural approach adopted by the Court suggests that it might avoid at least some of the pitfalls associated with some of its prior decisions, as well as with the judicialization of health policy-making in general [27,28]. For example, the Court does not assume it knows best what benefits should be included under the POS/POSS, nor the precise ethical grounds for making these determinations. Rather, in keeping with recent proposals in health ethics, the decision calls for a participatory process that is transparent, based on relevant reasons and current epidemiological information, subject to revision, and enforceable [29–31]. As it did in a similar structural order related to internally displaced persons (T-025/2004), the Court appears likely to adopt creative mechanisms for the supervision of this judgment, including public hearings with multiple stakeholders from government as well as civil society. However, in 2009 the Court's composition will change substantially, and it is unclear whether the new Court will assume the same degree of responsibility for overseeing the implementation of the judgment's complex structural orders.

Further, this decision, like most of the Court's other decisions, is directed at enforcing access to services, treatments, and medications. In the past, the Court has adopted some sweeping decisions with regard to public health measures, such as vaccination campaigns for poor children (SU-225/1998). Yet, paradoxically, the overall trend in judicial activism may in fact reinforce the effects of the 1993 health reform, which invested the majority of the health budget in individual insurance at the expense of public health promotion and prevention. Empirical investigation is needed to determine whether health resources are increased or merely shifted, what the effects on equity are, and whether the overall health system's infrastructure and workforce, as well as health promotion and prevention activities, are neglected as a result of policies stemming from the decision.

Yet, the impacts of the Court's activism in relation to the right to health should not be evaluated in isolation from its consideration of other economic and social rights, including housing and education—which are critical social determinants of health—as well as its progressive treatment with respect to gender and ethnic discrimination [32–34]. Further, grass-roots groups have found in the Court's jurisprudence a political banner that inspires them to use legal strategies to vindicate rights and seek social change [34]. Nonetheless, Colombia remains a profoundly unequal society, and there are clearly limits to the role of the Court in restructuring the fundamental social disparities that underlie many health inequalities.

## Conclusions

To a greater extent than in any other country, the Colombian Constitutional Court has exercised dramatic control over health policies and programming decisions. The Colombian example shows that increased access to courts may under certain circumstances enhance the protection of the right to health, as well as potentially promote equity and transparency in coverage definitions and greater accountability within the health system itself. However, further investigation is required with regard to the empirical effects of this judgment on the organization of an integrated health system, health budget-setting, and the availability, accessibility, acceptability, and quality of health facilities, goods, and services in Colombia.

## Supporting Information

Alternative Language Abstract S1 Spanish Translation of an Article Summary by AEY and OPV (23 KB DOC).

## Abbreviations

POS: *Plan Obligatorio de Salud*
POSS: *Plan Obligatorio de Salud Subsidiado*
