# Supplementary material for: How Do Courts Set Health Policy? The Case of the Colombian Constitutional Court
Source: PLoS Med. 2009 Feb 17;6(2):e1000032. doi: 10.1371/journal.pmed.1000032 (PMC2642877; doi:10.1371/journal.pmed.1000032)
Supplement: Supplementary file 1 [file pmed.1000032.sd001.doc]

Resumen: La protección judicial del derecho a la salud ha ido evolucionando en los países de bajos y medianos recursos, lo cual ha tenido implicaciones respecto a políticas públicas y gasto social. Existe un profundo debate en los estudios de salud pública en relación con la idoneidad de la intervención judicial frente a la política en salud y la equidad en salud, particularmente respecto a si el activismo judicial distorsiona prioridades y genera una intervención indebida en las competencias de los órganos administrativos y legislativos. En julio de 2008, la Corte Constitucional de Colombia emitió una sentencia (T-760 de 2008) que constituye un hito respecto a la intervención judicial con impacto en políticas públicas. El caso colombiano demuestra que el poder judicial no solo puede garantizar el suministro de bienes y servicios en salud, sino que también puede impulsar cambios estructurales del sistema de salud, planteando ello como problema de derechos fundamentales. Se necesita de investigaciones posteriores que analicen la implementación e impacto real de esta sentencia en la organización de un sistema integrado de salud, la destinación de recursos en salud y la disponibilidad, accesibilidad, aceptabilidad y calidad de instalaciones, bienes y servicios en Colombia. Con todo, el ejemplo de Colombia muestra que el aumento en el acceso a la justicia tiene un potencial para impulsar la protección del derecho a la salud bajo ciertas circunstancias.

Abstract: Health litigation is increasingly taking place in middle- and low-income countries, which has implications for policy and spending. There is a wide-ranging debate in public health circles about the appropriateness of judicial interventions on health policy and health equity, including whether such judicial activism distorts priority-setting and undermines the role of administrative and legislative bodies. In July, 2008, the Colombian Constitutional Court issued a decision (T-760/08), which breaks new ground with respect to what courts have done in relation to health policy-making. The Colombian case demonstrates that judiciaries can not only enforce the provision of health goods and services, but also require structural changes in the organization of the health system, as a matter of fundamental rights. Further investigation is required regarding the implementation and empirical effects of this judgment on the organization of an integrated health system, health budget-setting and the availability, accessibility, acceptability and quality of health facilities, goods and services in Colombia. Yet, the Colombian example shows that increased access to courts may potentially enhance the protection of the right to health under certain circumstances.
